# Evaluation of Lymphoproliferative Disease and Increased Risk of Lymphoma in Activated Phosphoinositide 3 Kinase Delta Syndrome: A Case Report With Discussion

**DOI:** 10.3389/fped.2018.00402

**Published:** 2018-12-18

**Authors:** Michele N. Pham, Charlotte Cunningham-Rundles

**Affiliations:** ^1^Division of Clinical Immunology, Department of Medicine, Icahn School of Medicine at Mount Sinai, New York, NY, United States; ^2^Division of Pulmonary, Critical Care, Allergy and Sleep Medicine, Department of Medicine, University of California, San Francisco, San Francisco, CA, United States

**Keywords:** activated phosphoinositide 3-kinase δ syndrome, phosphoinositide 3-kinase, immunodeficiency, activating mutations, hypogammaglobinemia, lymphoproliferative disease

## Abstract

Activated phosphoionositide-3 kinase delta syndrome (APDS) is a rare disorder caused by activating mutations in phosphoionositide 3-kinase delta (PI3Kδ). This syndrome usually presents in childhood with recurrent sinopulmonary infections and immune deficiency as is seen in the case discussed in this report. Patients with APDS also experience other complications including lymphoid hyperplasia, autoimmunity, increased susceptibility to herpes viruses, especially Epstein-Barr virus and cytomegalovirus, and an increased incidence of B-cell lymphoma. The clinical implications for lymphoid hyperplasia and lymphoma are profound and frequently, it is challenging to distinguish between the two. This case report is of a young girl with a mutation in PIK3CD, the gene encoding the catalytic subunit of PI3Kδ, who presents with asymmetrical cervical lymphadenopathy and parotid swelling. After little improvement in lymphadenopathy on antibiotics, an excisional biopsy of a cervical lymph node was obtained which was initially concerning for lymphoma. This case recounts the clinical decisions made to evaluate this lymphadenopathy and concern for malignancy due to the increased incidence of B-cell lymphoma in this population. It was concluded after careful evaluation of her lymph node histology and cytometry, bone marrow biopsy, and CSF studies that her findings were consistent with lymphoid hyperplasia and not lymphoma and she was treated with rituximab. This case highlights the many comorbidities present in patients with this disease and the current treatments for complications in patients with APDS, including new targeted therapies.

## Introduction

Balanced signaling in the PI3Kδ pathway is critical for normal immune function. Patients with activated phosphoionositide-3 kinase delta syndrome (APDS) characteristically have recurrent sinopulmonary infections, lymphadenopathy and lymphoid hyperplasia, as well as increased susceptibility to herpesviruses—Epstein-Barr virus (EBV) and cytomegalovirus (CMV) (1–5). These patients also have increased incidence of B-cell lymphomas and increased autoimmunity (1–5).

In this case report we will discuss the clinical course of a patient with an activating mutation in PI3Kδ who developed parotid and cervical swelling that prompted further evaluation and treatment. In addition to discussing lymphadenopathy in these patients, the manuscript will also review treatment approaches. Written informed consent was provided by her parent for the publication of this case report.

## Case Presentation

At the age of 2, our female patient was referred by pulmonary to clinical immunology for evaluation of her recurrent pneumonias and bronchiectasis. She had no family history suggestive of an immunodeficiency. Work-up at that time revealed IgA < 6 (34–305 mg/dL), IgG 30 (572–1,474 mg/dL), IgM 1190 (31–208 mg/dL). In addition to low levels of IgG and IgA but elevated IgM levels, immune evaluation revealed T and B cell lymphopenia −827 (876–3,394 CU MM) and 28 (200–1,259/CU MM), respectively. Her CD4 count was 331 (412–2,095/CU MM) and CD8 count was 481 (236–995/CU MM). She had a normal response to lymphocyte mitogen and antigen stimulation. Due to these results and her clinical history, she was started on intravenous immunoglobulin replacement which helped decrease her incidence of infections.

As she grew older she began to have increased hospitalizations for hemolytic anemia and recurrent pneumonias and sinusitis. She also developed lymphadenopathy and splenomegaly. She received rituximab with resultant improvement in her counts and decrease in size of her lymphadenopathy and hepatosplenomegaly. She was also started on trimethoprim/sufamethoxazole prophylaxis to help prevent infections, and though she continued to have intermittent respiratory infections the amount was improved.

Genetic testing revealed no mutation in AID, UNG, CD40, or CD40L but later gene sequencing discovered a dominant activating mutation in PIK3CD–c.3061G > A (p.Glu1021Lys). At the age of 10 she was started on rapamycin to help with her lymphoproliferative disease. Her compliance was inconsistent and she continued to have lymphadenopathy and intermittent declines in her blood cell counts Table 1.

**Table 1:** Clinical laboratory data overtime.

	**Normal range**	**1 year prior**	**2 months prior**	**1 month prior**	**Presentation**	**1 week after presentation**	**Before 4th^**d**^ rituximab dose**	**1 month after rituximab cycle**	**6 months after presentation**
White blood cell count	4.5–11.4 × 10 3/uL	4.2 (L)	5.0	3.5 (L)	3.1 (L)	2.5 (L)	4.4 (L)	4.4 (L)	3.5 (L)
Hemoglobin	10.6–14.4 g/dL	11.1	9.7 (L)	10.5 (L)	8.7 (L)	8.1(L)	9.1 (L)	9.3 (L)	12.3
Platelet	150–450 × 10 3/uL	101 (L)	84 (L)	78 (L)	74 (L)	50 (L)	112 (L)	94 (L)	75 (L)
Neutrophil count	1.8–7.8 × 10 3/uL	0.7 (L)	1.75 (L)	1.4 (L)	1.4 (L)	1.0 (L)	1.7 (L)	1.8	1.7 (L)
Lymphocyte count	1.0–3.5 × 10 3/uL	2.6	2.95	2.0	1.3	1.1	2.2	2.1	1.5
Lactate dehydrogenase	100–220 u/L	176	200	181	192	147	-	156	176

At 11 years of age, she presented to clinic with 1 week of asymmetrical (left greater than right) cheek swelling that was not tender to palpation. An ultrasound revealed diffusely enlarged bilateral parotid and submandibular glands containing numerous hypoechoic nodules and foci of increased echogenicity. Imaging also showed bilateral cervical lymphadenopathy, again left greater than right. She initially was treated with oral clindamycin but continued to have fevers so was admitted for intravenous (IV) antibiotics. After a 5-day admission with IV clindamycin and piperacillin/tazobactam treatment the swelling did not improve so a lymph node biopsy was pursued.

**Figure 1:**
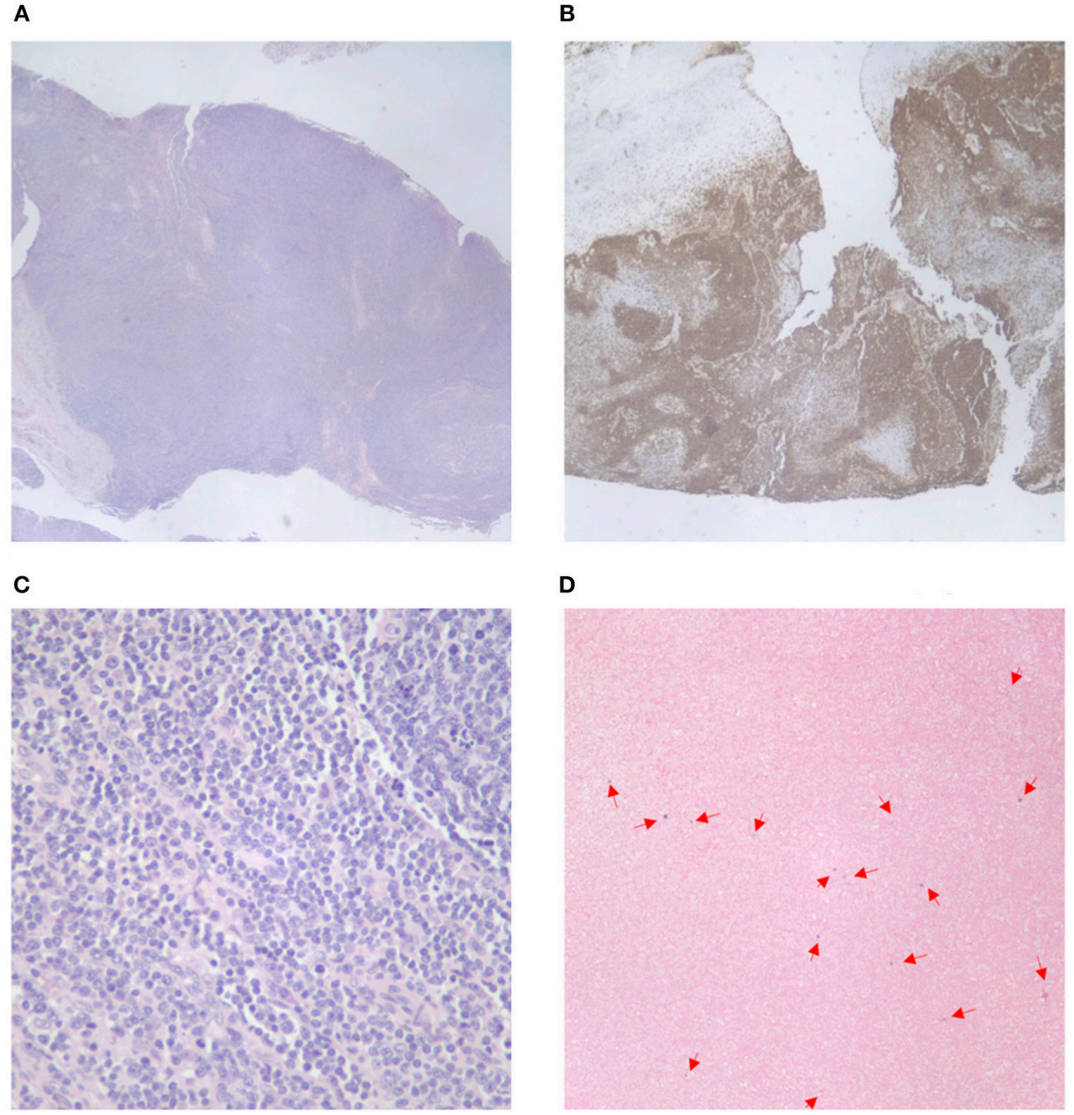
Cervical lymph node pathology. **(A)** Effacement of lymph node architecture, with vague nodular growth pattern. **(B)** Increased CD20 tissue staining in the lymph node tissue. **(C)** Increased CD20 tissue staining in the lymph node tissue (40x magnification). **(D)** EBV *in situ* hybridization—positive staining for EBV in a small amount of scattered lymphoid cells.

**Figure 2:**
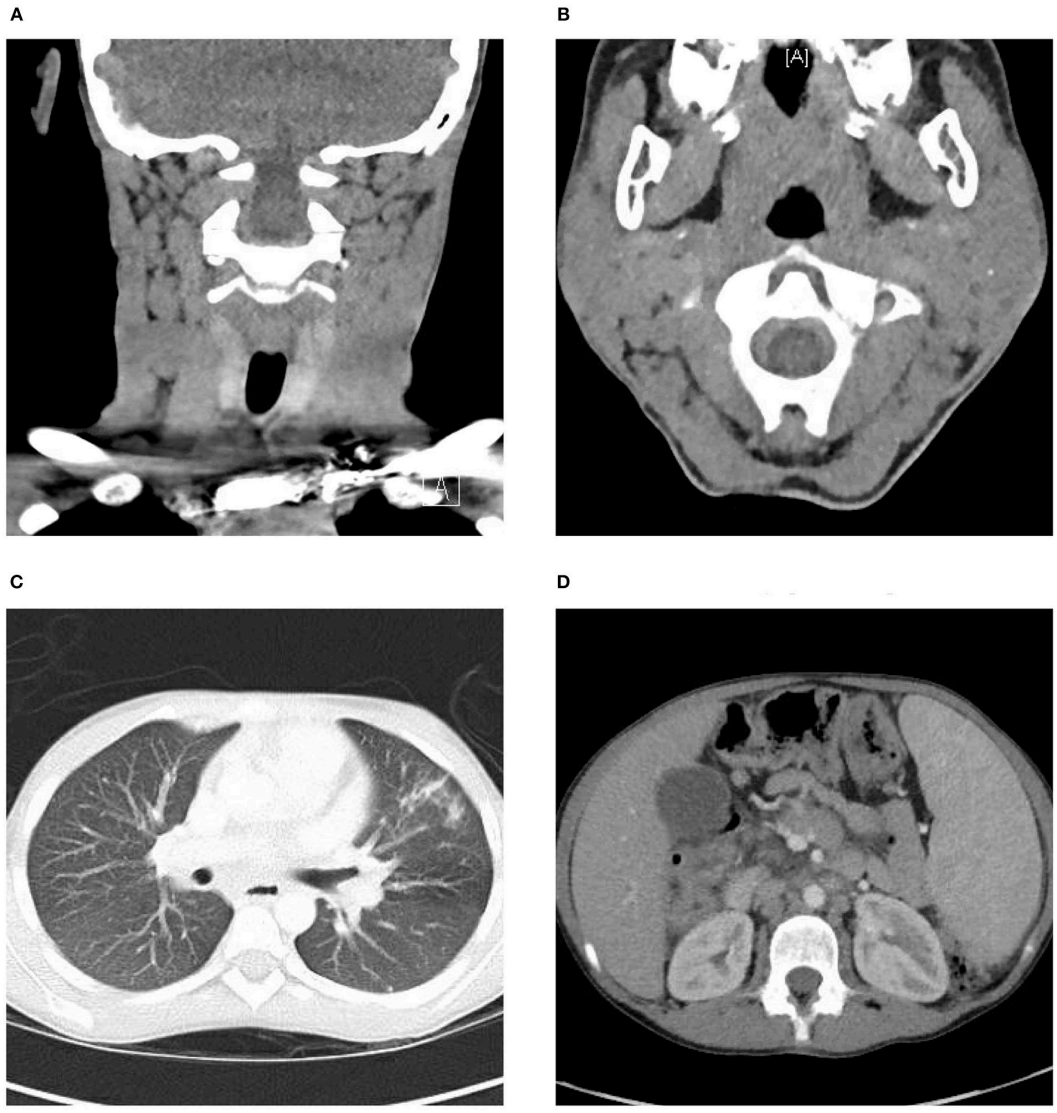
CT head and neck, chest, abdomen, and pelvis. **(A)** CT demonstrating left greater than right scattered cervical lymphadenopathy. **(B)** Bilateral parotid gland enlargement, left greater than right. **(C)** Mediastinal and hilar lymphadenopathy, multiple calcified and noncalcified lung nodules, lung cysts, and bronchiectasis. **(D)** Hepatosplenomegaly, abdominal and pelvic lymphadenopathy as well as marked thickening of the distal through terminal ileum wall and the wall of the cecum extending up to the hepatic flexure.

An excisional biopsy of a left cervical lymph node revealed initial findings concerning for malignancy due to lymph node architecture effacement Figure 1A and increased CD20 staining Figures 1B,C as well as B cell clonality on flow cytometry (31–33% of the B cell population was aberrant exhibiting CD45+, CD19+, CD20+, CD5–, CD10–, CD23dim+, CD22+, FMC7 bright+, sIg Kappa dim+). Since she had received corticosteroids which could partially treat a lymphoma she underwent a bone marrow biopsy and cerebral spinal fluid evaluation to ensure adequacy of workup and to evaluate for possible disease extent. In addition to asymmetrical cervical lymphadenopathy and parotid gland enlargement Figures 2A,B, a CT neck/chest/abdomen and pelvis done at that time showed mediastinal, hilar, abdominal, and pelvic lymphadenopathy, impressive retroperitoneal lymphadenopathy, hepatosplenomegaly, and cecum and terminal ileum thickening Figures 2C,D.

A few days later her left cervical lymph node biopsy and her bone marrow biopsy results were finalized. Her bone marrow biopsy showed a normocellular marrow with no significant dysplasia or expansion of blasts. Further molecular testing of her cervical lymph node biopsy was negative for IgH/IgK, TCR alpha/beta, and TCR gamma/delta gene rearrangements and there were no aberrant T cells. Follicles with germinal centers were positive for CD20, CD79a, CD23, and BCL6. Staining revealed small scattered EBV positivity in lymphoid cells with a polymorphic population of T cells, B cells, and blasts Figure 1D. These findings are similar to EBV-associated polymorphous lymphoproliferative disorders seen in immunosuppressed post-transplant patients. This lymphoproliferative disorder has multiple stages that range from early lesions and polymorphic disease to monomorphic disease and lymphoma (6).

Despite the initial concerning features, due to her known immunodeficiency, clinical presentation, findings of a normal bone marrow, and lymph node tissue findings suggestive of an EBV-associated polymorphous lymphoproliferative disease, it was concluded that she did not have lymphoma. She was diagnosed with polymorphous B-cell lymphoproliferative disorder in the setting of primary immune deficiency disorder with clonal B cells by flow cytometry findings. She began treatment with rituximab and her cervical lymphadenopathy as well as parotid gland swelling greatly improved.

## Discussion

### Phosphoinositide-3-Kinase δ

Phosphoinositide-3-kinase δ (PI3Kδ) is a heterodimer comprised of the phosphatidylinositol-4,5-bisphosphate 3-kinase catalytic subunit delta isoform (p110δ) and a p85 family regulatory subunit. PI3Kδ is present predominantly in leukocytes and plays an important role in leukocyte proliferation, activation, and survival (2). Its catalytic subunit p110δ is encoded by the PIK3CD gene.

Immunodeficiency caused by activating mutations in PI3KCD are referred to by the names activated phosphoionositide-3 kinase delta syndrome (APDS) and p110δ activating mutation causing senescent T-cells, lymphadenopathy and immunodeficiency (PASLI) (5). The GLU1021LYS (E1021K) mutation in the catalytic subunit results in a gain of function and is the most common mutation found in this disorder (1, 2, 5). Other described mutations that lead to APDS are ASN334LYS (N334K), GLU525LYS (E525K), and CYS416ARG (C416R) (2–5). This condition has an autosomal dominant inheritance pattern and these mutations enhance membrane association, PIP3 production, and PI3K-Akt pathway activation as well as excessive activation of mTOR (2, 5).

Subsequently it was found that PI3Kδ activation also results from splice site mutations of the PIK3R1 gene which encodes for the p85α subunit. Binding of p85α subunit to p110δ inhibits its activity (7, 8). These patients have a similar immunological phenotype to those with PIK3CD mutations but have more systemic features including neurodevelopmental delay, growth retardation, and autoimmunity (7, 8). These disorders are termed APDS 2 (activated phosphoinositol kinase delta syndrome, type 2 (P85 mutations) or PASLI-R1 (OMIM 616005) (7, 8). Other mutations, for instance ones that cause hyper-IgM syndrome, also result in similar clinical phenotypes and lab findings of low IgA and IgG but normal/elevated IgM. As in our case, when CD40/CD40L and AID/UNG testing are unrevealing in a patient with immune deficiency, normal/elevated IgM, recurrent infections, and lymphadenopathy, activating PI3Kδ mutations should be considered (3, 5).

### Clinical Manifestations

Recurrent infections—particularly of the lungs, sinuses, and ears—in these patients usually begin in childhood (1). In a study of 53 patients with PI3Kδ mutations, the most common infectious presentations were recurrent respiratory tract infections (98%) and pneumonia (85%) (1). In the literature, 33–75% of APDS patients have bronchiectasis (1, 2). The most common bacterial pathogens are *Streptococcus pneumoniae* and *Haemophilus influenzae* (1). Persistent, severe or recurrent herpesvirus infections, particularly EBV, CMV, and varicella zoster virus, were found in one study to be present in 49% of patients (1). Adenovirus, warts, and molluscum contagiosum were also reported (1). Additionally, in this study 13% had oral mucocutaneous candidiasis requiring treatment (1). Autoimmune manifestations are also frequently described and include cytopenias and exocrine pancreatic insufficiency that are experienced by our patient (1).

### Lymphoproliferative Complications

It is believed that activation of PI3Kδ signaling stimulates abnormal proliferation of white blood cells and leads to lymphadenopathy and nodular lymphoid hyperplasia (4). Non-neoplastic lymphoproliferation, thus, is a common comorbidity and has been reported to be up to 75% of these patients with lymphadenopathy (64%), splenomegaly (58%), or hepatomegaly (45%) being the more common manifestations for disease (1). In this population, nodular mucosal lymphoid hyperplasia was found in 32% and having lymphadenopathy was significantly associated with mucosal lymphoid hyperplasia (OR, 16; 95% CI, 1.9–133.8; *P* = 0.002), splenomegaly (OR, 9.1; 95% CI, 2.5–33.2; *P* = 0.0005), and herpesvirus infection (OR, 6.9; 95% CI, 1.9–25.2; *P* = 0.004) (1). Though these changes are benign, complications may ensue depending on the site of involvement and the increase in B-cell proliferation in combination with a reduced immune system may contribute to the development of B-cell lymphoma (3, 4).

Lymphoma has been reported in about 13% of APDS patients (1). Other theories suggest that the increased risk of malignancy in APDS may be due to the inherent genetic instability of lymphocytes and the persistent lymphoid system activation in response to infections, autoimmune disease and immune deficiency (9). EBV-infection is found in about 30% of APDS patients and highlights the important role of PI3Kδ signaling in controlling EBV through B and T cell development and function (10). Polymorphous B-cell lymphoproliferative disorder, as seen in our immunodeficient patient, is part of a spectrum of lymphoid proliferations that are typically EBV-associated (6). In the literature, lymphoid hyperplasia and lymphomas in patients with APDS have variable association with EBV. Thus, there are likely other mechanisms and a wider spectrum of PI3Kδ cancer susceptibility not yet clearly uncovered (3, 4, 10). The PIK3CD gene has been characterized as an oncogene associated with diffuse large B-cell lymphomas, and constitutive PI3K activation has also been linked to other B-cell malignances, e.g., Burkitt lymphomas (4, 11).

### Evaluation for Lymphoproliferative Disease and Lymphoma

As discussed, lymphadenopathy is common in APDS patients and the evaluation of this is challenging due to the broad differential (1, 3, 4, 12). When lymphadenopathy appears to persist or worsen despite treatment, further examination may be required to evaluate for malignancy, especially in the setting of constitutional symptoms. It is important to also note that lymphomas may be nodal and/or extranodal (9). This poses challenges since, for example, patients may have lymphoid hyperplasia in MALT-associated sites making it difficult to distinguish between benign and malignant proliferations (9).

Histologically, lymphomas tend to exhibit extensive growth that leads to compression of interfollicular space or loss of prior compartments (13). Follicular lymphomas are more likely to have findings such as B cell overexpression of BCl2 than tissues with benign lymphoid hyperplasia (13). In our patient, positive EBV lymph node staining as well as a polymorphic cell population and clinical history helped provide clues to distinguish her polymorphous B-cell lymphoproliferative disorder from malignancy. The presence of a monoclonal process on histology may aid in diagnosis of a malignant process but clonal lymphocyte populations may also be seen in patients without lymphoma (12). Further evaluation with immunohistochemical and gene rearrangement studies are particularly helpful to determine cell lineage as well as to detect oncogene/chromosomal translocation (12).

Astute and experienced clinicians and pathologists are needed to further distinguish between benign lymphoproliferative disease and lymphoma. The final diagnosis arises after taking into account the patient's clinical presentation and history, the histological appearance of the tissue, and presence of characteristic markers. Making a distinction is important since treatment plans will differ.

### Treatment of Lymphoproliferative Disease in APDS

At this time, the only available curative option for APDS is hematopoietic stem cell transplantation (HSCT) which has been performed for a small number of cases with some successes in restoring normal growth and resolution of infection and non-neoplastic lymphoproliferation (14, 15). In one report, 9 of 11 patients who received HSCT survived at 8 or greater months post-transplant (15). Some complications seen included poor engraftment, chronic graft-versus-host-disease and infection (14, 15).

Other therapies aim to manage the comorbidities associated with this syndrome, notably the infectious, autoimmune, and lymphoproliferative complications Table 2. Treatment focuses on providing defenses for the immune system and addressing dysregulation. Antimicrobial prophylaxis and immunoglobulin replacement have a reported benefit with decreasing infections (1, 4, 5). For some, management of autoimmune complications and lymphoproliferative disease also necessitates the use of corticosteroids (3, 16). In a European ESID-APDS-registry with 77 patients (51 APDS1, 26 APDS2), 31 patients received corticosteroids and 27 received a partial clinical effect, with more than half of the patients receiving treatment by the age of 20 (16). It is noted, though, that corticosteroids do pose many undesirable side effects such as elevated blood sugars and increased infections.

**Table 2:** Therapies used in patients with APDS.

	**Mechanism of action**	**Affects**	**Findings in APDS patients**
Immunoglobulin replacement (4, 5)	Replacement of IgG for antibody protection	Infectious complications Autoimmunity (e.g., hemolytic anemia, ITP)	Decrease in infections, stabilization of platelet and hemoglobin counts
Antibiotic prophylaxis (4, 5)	Anti-microbial	Infectious complications	Decrease in infections
Steroids (16)	Anti-inflammatory, immune modulation	Lymphoproliferation, Autoimmunity (e.g., hemolytic anemia, ITP)	Decreased inflammation, decrease in lymphadenopathy, stabilization of blood cell counts
Rituximab (1, 16)	Monoclonal CD20 antibody	Lymphoproliferation, Autoimmunity (e.g., hemolytic anemia, ITP)	Improvement in lymphoproliferative disease and stabilization of blood cell counts
Rapamycin (2, 8, 16)	mTOR inhibitor T cell directed therapy	Lymphoproliferation, Colitis, Autoimmunity (e.g., hemolytic anemia, ITP)	Diminished ribosomal protein S6 phosphorylation, improvement in lymphoproliferative disease, stabilization of blood counts.
Leniolisib (CDZ173) (17)	Small molecule inhibitor of PI3K catalytic subunit p110δ	Lymphoproliferation, immune deficiency	Decrease in lymphadenopathy, reduction in senescent CD4 and CD8 T cells, decrease in transitional B cells and normalization of naïve B cells. Dose dependent reduction in AKT and ribosomal protein S6 phosphorylation.
Hematopoietic stem cell transplantation (14, 15)	Reestablishment hematopoietic function	Potentially curative of APDS	Performed for a small number of cases with some successes in restoration of normal growth and resolution of infection and non-neoplastic lymphoproliferation.

Rituximab has been shown to be of benefit in management of autoimmune hemolytic anemia and non-neoplastic lymphoproliferative disease (1, 16). This monoclonal CD20 antibody has been used with response in our patient and in multiple other APDS patients with clinical benefit, though some did experience sustained B-cell lymphopenia (1, 16). Other immunosuppressive drugs including azathioprine, mycophenolate, cyclosporine, and rapamycin have also been used (1, 2, 16).

Rapamycin (sirolimus), a medication commonly used to prevent transplant rejection, has been shown to effectively reduce hepatosplenomegaly and lymphadenopathy in APDS and other immune deficiency patients (1, 2, 16). In patients with activating mutations in PI3KCD, functional testing demonstrates increased AKT phosphorylation and mTOR signaling (2, 5). With the use of rapamycin, an mTOR inhibitor, lymphocyte activation is inhibited and *in vitro* studies have demonstrated diminished ribosomal protein S6 phosphorylation (2). In one cohort study with 53 APDS patients, 6 patients underwent treatment with rapamycin for benign lymphoproliferation with 5 having a response (1). The remaining patient had to stop treatment due to side effects. In the ESID-APDS-registry cohort, rapamycin was the most frequently prescribed immunosuppressive medication and was used in 26 patients (17 with APDS1 and 9 with APDS2) most commonly for lymphoproliferative disease, colitis, or cytopenia (16). In this cohort, lymphoproliferative disease responded the best with 8 patients having complete resolution, 11 showing partial responses, and 6 experiencing no remission. Bowel inflammation and cytopenias did not respond as well to treatment with rapamycin (16). Five patients had to stop rapamycin due to side effects such as severe headaches, anorexia, and renal toxicity (16). Of the 8 patients on steroids at the start of rapamycin, 7 were able to stop and 1 was able to reduce the dose (16).

Targeted therapies have also increased in the last few years. A method of treatment in APDS patients includes addressing the dysregulation in PI3Kδ. There are selective p110δ inhibitors, such as GS-1101 (CAL-101 or idelalisib), that have been studied in patients with chronic lymphocytic leukemia and these drugs have been tolerated suggesting that this treatment may prevent lymphoma development (4, 5). Leniolisib (CDZ173), a small molecule inhibitor of p110δ, was found in a clinical trial of 6 APDS patients to cause dose-dependent suppression of PI3Kδ pathway hyperactivation (17). In this trial, the oral medication improved lymphoproliferation—lymph node sizes and spleen volumes were reduced by 39 and 40%, respectively, (17). Levels of senescent CD4 and CD8 T cells as well as transitional B cells were reduced (17). The medication was also overall well tolerated with no significant laboratory disturbances or clinical side effects noted (17). The development of medications such as Leniolisib has increased options for targeted therapies and allowed for the increased practice of precision-medicine.

## Conclusion

Activating mutations in PI3Kδ have been recently discovered in the past few years due to the advances in sequencing and genetic evaluation of primary immunodeficiencies. Having access to these tests allow for the discovery of a molecular cause of immune system dysregulation and allows clinicians to tailor evaluations to known comorbidities, for instance an increased incidence of lymphoproliferative disease. Knowing these molecular abnormalities also helps guide therapy selection and as physician-scientists learn more about the immune system, gene alterations, and resultant phenotypes, targeted therapies may be an increased avenue for treatment.

## Author Contributions

MP: wrote the manuscript. CC-R: critically reviewed the manuscript.

### Conflict of Interest Statement

The authors declare that the research was conducted in the absence of any commercial or financial relationships that could be construed as a potential conflict of interest.
